# Supplementary material for: Bone Growth Induction in Mucopolysaccharidosis IVA Mouse
Source: Int J Mol Sci. 2023 Jun 8;24(12):9890. doi: 10.3390/ijms24129890 (PMC10298227; doi:10.3390/ijms24129890)
Supplement: Supplementary file 1 [file ijms-24-09890-s001.zip › ijms-2378747-supplementary.pdf]

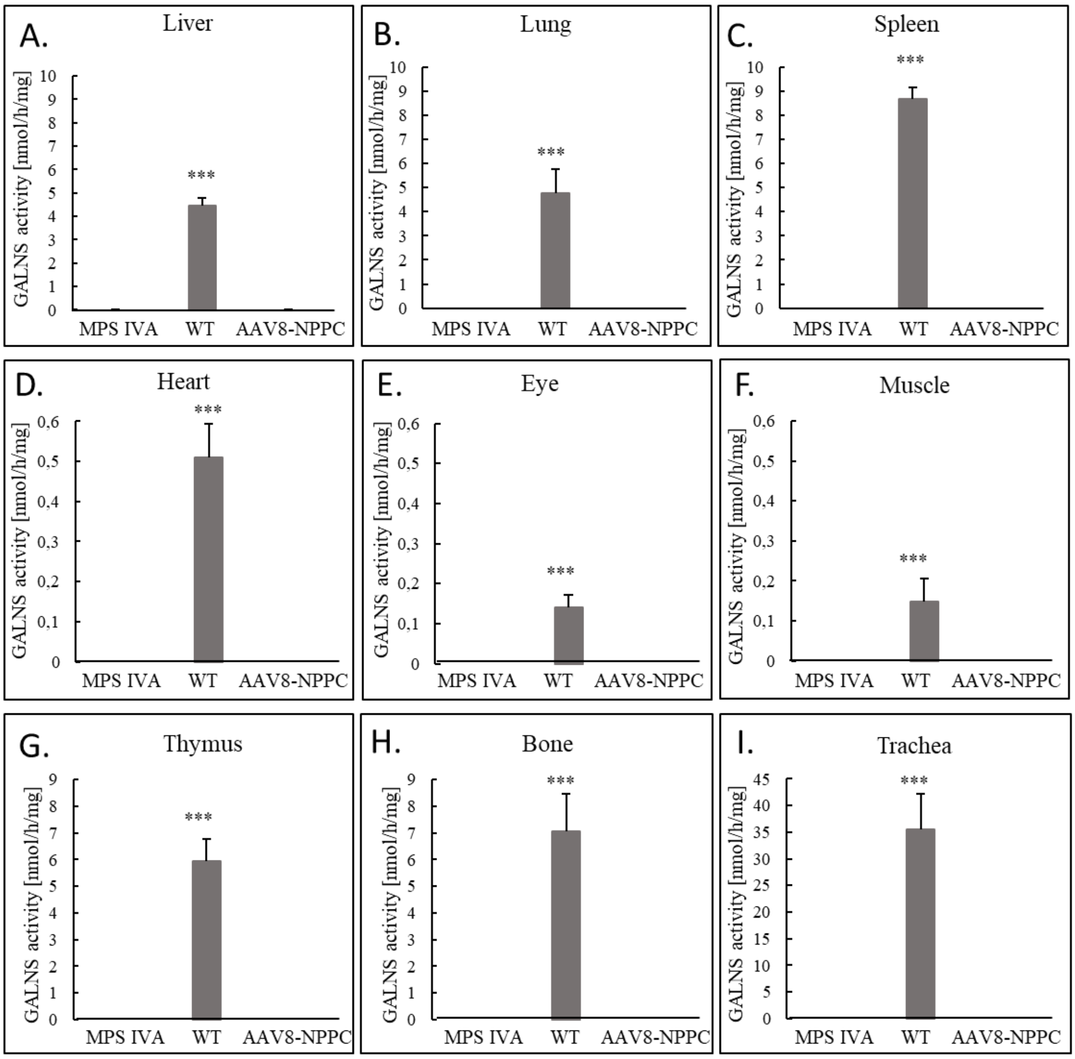

Figure S1. GALNS enzyme assay activity. After 12 weeks post-treatment, we measured GALNS enzyme assay activity in tissues: liver (A), lung (B), spleen (C), heart (D), eye (E), muscle (F), thymus (G), bone (H), trachea (I). Results are shown as mean values ± SEM (n=5). The following statistical symbols were used to denote as follows. AAV8-CNP group vs. WT group, *** $p \leqslant 0.001$.

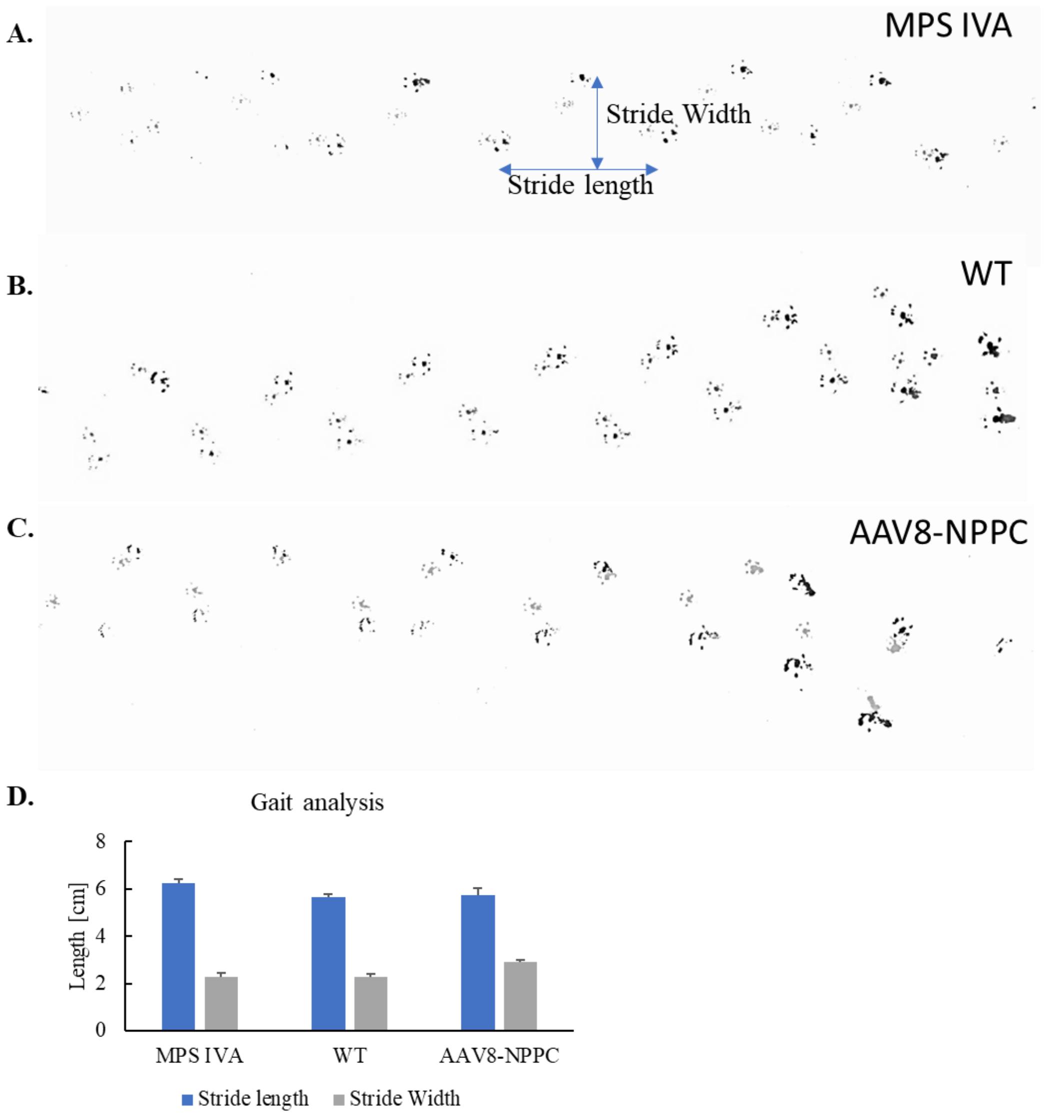

Figure S2. Gait analysis. To analyze the walking pattern of mice, we performed a gait analysis on the final week before the autopsy. Representation of the walking pattern of MPS IVA (A), WT (B), and AAV8-NPPC (C), calculation of stride length and width (D). Results are shown as mean values ± SEM (n=5). No significant difference was observed between the groups.

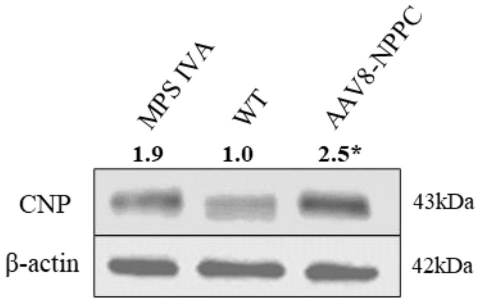

Figure S3. CNP protein expression in the liver tissue. Relative levels of the proteins were measured using the Western-blotting procedure. Representative blots are shown and data were quantitated by densitometry. The following statistical symbols were used to denote as follows. AAV8-CNP group vs. WT group, * $p \leqslant 0.05$.

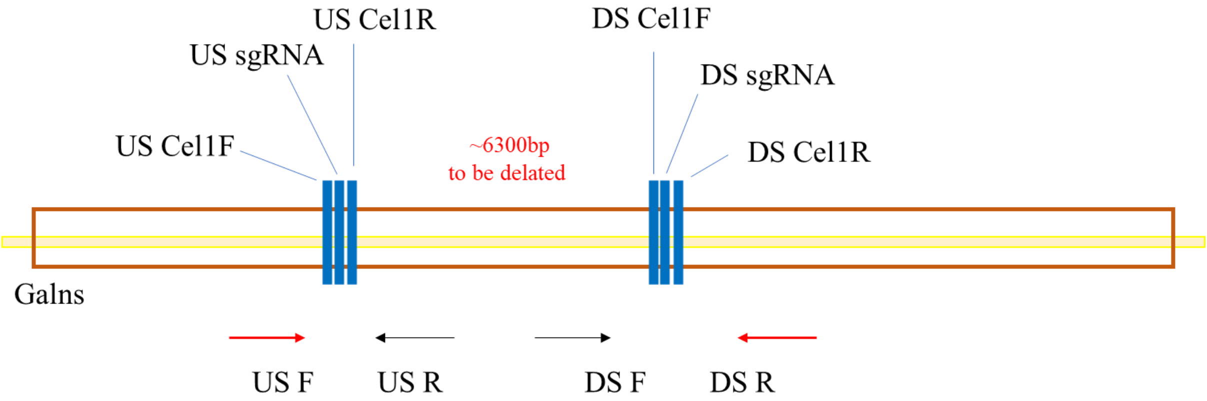

Figure S4. Construct of the GALNS gene deletions. Two pairs of sgRNAs cleaved together to generate a large deletion between two target sites. Primers flanking each sgRNA site were designed to test individual NHEJ activity, as well as paired together (red arrows) to screen for deletion mutations between the two target sites. All mutations were sequence verified. US-upstream, DS-downstream
